# Long-Term Outcomes of Local Excision Following Neoadjuvant Chemoradiotherapy for Locally Advanced Rectal Cancer

**DOI:** 10.1245/s10434-020-09243-6

**Published:** 2020-10-30

**Authors:** Lucrezia D’Alimonte, Quoc Riccardo Bao, Gaya Spolverato, Giulia Capelli, Paola Del Bianco, Laura Albertoni, Antonino De Paoli, Mario Guerrieri, Giovanna Mantello, Maria Antonietta Gambacorta, Vincenzo Canzonieri, Vincenzo Valentini, Claudio Coco, Salvatore Pucciarelli

**Affiliations:** 1 Department of Surgical, Oncological, and Gastroenterological Sciences, First Surgical Clinic, University of Padua, Padua, Italy; 2 Clinical Research Unit, Istituto Oncologico Veneto IOV – IRCCS, Padua, Italy; 3 Surgical Pathology and Cytopathology Unit, Department of Medicine-DIMED, University of Padua, Padua, Italy; 4 Department of Radiation Oncology, National Cancer Institute, Aviano, Italy; 5 General Surgery, Marche Polytechnic University, Ancona, Italy; 6 Department of Radiotherapy, State Hospital, Ancona, Italy; 7 Department of Radiotherapy, Catholic University of Rome, Rome, Italy; 8 Department of Pathology, National Cancer Institute, Aviano, Italy; 9 Department of Surgical Sciences, Catholic University of Rome, Rome, Italy

## Abstract

**Background:**

Local excision might represent an alternative to total mesorectal excision for patients with locally advanced rectal cancer who achieve a major or complete clinical response after neoadjuvant chemoradiotherapy.

**Methods:**

Between August 2005 and July 2011, 63 patients with mid-low rectal adenocarcinoma who had a major/complete clinical response after neoadjuvant chemoradiotherapy were enrolled in a multicenter prospective phase 2 trial and underwent transanal full thickness local excision. The main endpoint of this study was to evaluate the 5- and 10-year overall, relapse-free, local, and distant relapse-free survival, which were calculated by applying the Kaplan–Meier method. The rate of patients with rectum preserved and without stoma were also calculated.

**Results:**

Of 63 patients, 38 (60%) were male and 25 (40%) were female, with a median (range) age of 64 (25–82) years. At baseline, the following clinical stages were found: cT2, *n* = 21 (33.3%); cT3, *n* = 42 (66.6%), 39 (61.9%) patients were cN+. At a median (range) follow-up of 108 (32–166) months, the estimated cumulative 5- and 10-year overall survival, relapse-free survival, local recurrence-free survival, and distant recurrence-free survival were 87% (95% CI 76–93) and 79% (95% CI 66–87), 89% (95% CI 78–94) and 82% (95% CI 66–91), both 91% (95% CI 81–96), and 90% (95% CI 80–95) and 86% (95% CI 73–93), respectively. Overall, 49 (77.8%) patients had their rectum preserved, and 54 (84.1%) were stoma-free.

**Conclusion:**

In highly selected patients, the local excision approach after neoadjuvant chemoradiotherapy is associated with excellent long-term outcomes, high rates of rectum preservation and absence of permanent stoma.

The standard of care for locally advanced rectal cancer is neoadjuvant chemoradiotherapy followed by total mesorectal excision (TME). This strategy has been shown to decrease the rate of local recurrence up to 6%^1^ with estimated 5- and 10-year overall survival (OS) of approximately 75 and 60%,^1^,^2^ respectively. Moreover, after neoadjuvant chemoradiotherapy, up to 28% of patients show a pathological complete response (pCR),^3^ and a further 20% show a major (few residual cancer cells) pathological response.^4^ These findings are clinically relevant as patients with pCR to neoadjuvant chemoradiotherapy followed by TME show a significantly better outcome compared with non-responders.^5^ However, TME is associated with higher rates of morbidity, impairment of bowel function and quality of life,^6^ and permanent stoma.

The above considerations explain the increasing interest in rectum-sparing approaches, either local excision (LE) or watch-and-wait, for patients with complete or major clinical response to neoadjuvant chemoradiotherapy. The transanal LE approach has been used for many years in patients unfit or refusing major surgery and has been evaluated by several retrospective studies. More recently, a few prospective trials have been performed in the setting of a rectum-sparing strategy for patients with both major and complete clinical response.^7^–^11^ These studies found that patients who underwent LE had comparable short-term oncological outcomes, associated with better bowel function and quality of life,^12^ and reduced rates of complications compared with those who underwent standard TME.^10^,^13^ While these studies report on short-term outcomes, there is a lack of information related to the long-term outcomes.^14^,^15^

The present study aimed to evaluate long-term survival outcomes of rectal cancer patients who underwent LE following neoadjuvant chemoradiotherapy.

## Methods

### Study Design

This study is an update of a previously published prospective multicenter phase 2 trial that enrolled patients who underwent LE after neoadjuvant chemoradiotherapy in four Italian centers.^8^ The trial was approved by the local ethics committee of each center involved in the study. Inclusion criteria, and patients, tumor, and treatment characteristics have been previously reported.^8^ The inclusion criteria were: age > 18 years, histologically confirmed rectal adenocarcinoma, located up to 11 cm from anal verge. Either clinical T3 or low-lying T2 tumors, with a major (complete or near complete) clinical response to neoadjuvant chemoradiotherapy were included. The baseline work-up included clinical history, digital rectal examination, colonoscopy, carcino-embryonic antigen level, chest/abdomen computed tomography scan and pelvic magnetic resonance imaging (MRI). Major clinical response has been previously defined as the absence of a positive regional lymph node on MRI, and either no mucosal abnormality or a flat residual scar (complete response), or a superficial ulcer less than 2 cm at proctoscopy (major response).^16^ The differentiation between complete and major clinical response was not formally required when the protocol was planned. Clinical and pathological TNM staging were reported according to the American Joint Committee on Cancer, 8th Edition.^17^ The following histopathological data were collected: yT stage, tumor regression grade (TRG),^18^ status of resection margin, tumor differentiation, and vascular and lymphatic invasion. The pathologic response was defined as complete (pCR) if no viable tumor cells were found in the surgical specimen. When residual cancer was found, the pathologist was requested to report on the presence of the following unfavorable features: pT > 1, lympho-vascular invasion, poor differentiation grade, involved margin, and TRG > 2.

### Treatment Details

Fluoropyrimidine-based chemotherapy was administered concomitantly with radiotherapy at a total dose of 50.4 Gy, given in 28 fractions of 1.8 Gy each. Patients were re-staged at least 5 weeks after the completion of neoadjuvant chemoradiotherapy and those with major response were considered eligible for LE and enrolled in the study. After signing the informed consent, the patients underwent a full-thickness excision using the traditional transanal approach or transanal endoscopic microsurgery (TEM).

While patients with pCR (ypT0), or with ypT1 and histopathologically favorable features were observed, those with residual cancer showing at least one of the unfavorable histopathological features were recommended to undergo a subsequent completion TME surgery.

### Long-Term Outcomes Definition and Statistical Analysis

Patients underwent a strict follow-up, the modalities of which have been detailed elsewhere.^8^ Local recurrence was defined as any pelvic endoluminal or extraluminal recurrence, while recurrences outside the pelvis were defined as distant. To evaluate the overall survival (OS), relapse-free survival (RFS), local relapse-free survival (LRFS), and distant relapse-free survival (DRFS), the Kaplan–Meier method was used. Each outcome was calculated from the date of LE to the date of the event (local, and distant recurrence, death, or the last follow-up). The proportion of patients without stoma or with rectum preserved were also calculated. All analyses were carried out with STATA version 13.0 (StataCorp, College Station, TX).

## Results

### Patient, Tumor, and Treatment Characteristics

Between August 2005 and July 2011, 63 patients were enrolled in the study. The baseline characteristics of patients, tumor, and treatment modality have been previously reported^8^ and are summarized in Table 1.

**Table 1:** Patient, tumor, and treatment characteristics

Variables	*N* = 63	%
Age	Median (range), years	64 (25–82)
*Gender*		
Males	38	60.3
Females	25	39.7
*Carcinoembryonic antigen level*		
≤ 5 ng/ml	37	58.7
> 5 ng/ml	7	11.1
Missing	19	30.2
*Baseline clinical T stage*		
cT2	21	33.3
cT3	42	66.6
*Baseline clinical N stage*		
Negative	24	38.1
Positive	39	61.9
Tumor distance from anal verge	Median (range), cm	5.8 (3–11)
*Radiotherapy, total dose*		
< 50.4 Gy	2	3.2
> 50.4 Gy	61	96.8
*Chemotherapy*		
Capecitabine or 5-fluorouracil alone	37	58.7
Capecitabine or 5-fluorouracil, and oxaliplatin	24	38.1
Others	2	3.2
*Local excision technique*		
Transanal excision	30	47.6
Transanal endoscopic microsurgery	33	52.4

### Histopathology

**Fig. 1:**
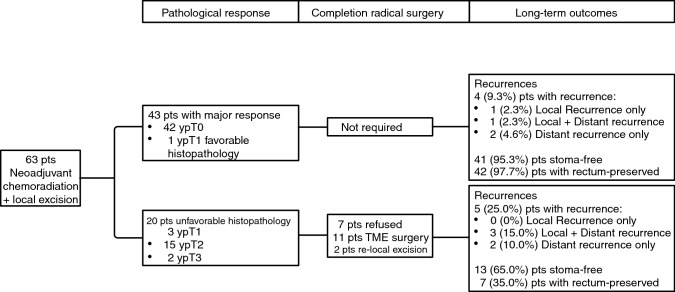
Pathological response, completion radical surgery and long-term outcomes of the study group after a median follow-up of 108 months

The histopathological tumor characteristics and post-LE treatment details are summarized in Table 2. Interestingly, out of 42 patients who showed a pCR, only 19 (45.2%) were clinically considered to be complete responders (ycT0), the remaining 23 (54.8%) had a major clinical response which was considered near complete. Therefore, on the basis of favorable histopathological features in one patient, a total of 43 patients were observed. A completion radical surgery was recommended in the remaining 20 patients. Of them, 11 underwent a TME (low anterior resection, *n* = 7; abdominoperineal resection, *n* = 4), two underwent a transanal local re-excision, and seven refused any further surgery (Fig. 1).

**Table 2:** Histopathology characteristics of tumors after local excision, and subsequent surgical treatment of patients with unfavorable histopathology features

Variables	*N* = 63	%
*ypT stage*		
T0	42	66.7
T1	4	6.3
T2	15	23.8
T3	2	3.2
*Tumor regression grade*		
1	42	66.7
2	5	7.9
3–5	16	25.4
*Margin*		
Negative	59	93.7
Positive	4	6.3
*Subsequent completion radical surgery*		
Low anterior resection	7	11.1
Abdominoperineal resection	4	6.3
Local re-excision^§^	2	3.2
Refused	7	11.1
Not required	43	68.3

^§^Patients refused major surgery and only accepted minor (local excision) surgery

### Outcomes

**Fig. 2:**
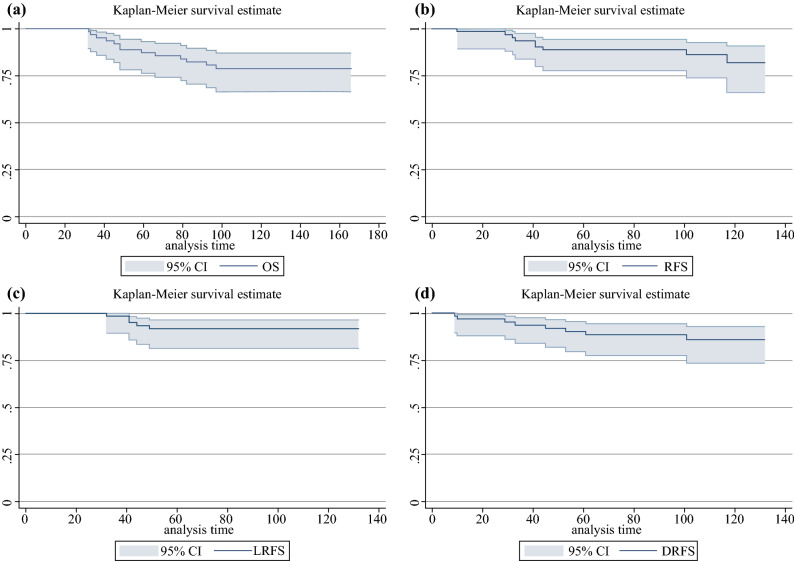
Kaplan–Meier estimate. **a** Overall survival. **b** Relapse-free survival. **c** Local relapse-free survival. **d** Distant relapse-free survival

Long-term survival outcomes are summarized in Fig. 2. At a median (range) follow-up of 108 (32–166) months, the estimated cumulative 5- and 10-year OS were 87% (95% CI 76–93) and 79% (95% CI 66–87), respectively (Fig. 2a). The estimated cumulative 5- and 10-year RFS were 89% (95% CI 78–94) and 82% (95% CI 66–91), respectively (Fig. 2b).

The estimated cumulative 5- and 10-year LRFS were both 91% (95% CI 81–96) (Fig. 2c). Overall, five (8%) patients were found to have a local recurrence, which occurred within the first 5 years of follow-up. Two recurrences were endoluminal and three extra-luminal. Characteristics of patients with local and distant recurrence are summarized in Table 3. Of five patients experiencing local recurrence, one refused further treatments, one underwent chemoradiotherapy for concomitant distant disease, and three underwent surgery (local re-excision, abdominoperineal resection, and low anterior resection + intraoperative radiotherapy). Of these five patients, four also showed distant recurrence and four died.

**Table 3:** Characteristics of tumors and patients who showed recurrence

Age/Gender	Baseline cTN	nCRT	Post nCRT ycTN	Post LE ypT	TRG	Margin	Completion TME surgery	TTLR (months)	Treatment for local recurrence	TTDR	Site of Distant recurrence
53/M	T3N+	Capecitabine + 50.4 Gy	ycT1N0	2	3	Negative	Required/Refused	49	Local re-excision	10	Liver
74/M	T3N+	Capecitabine + oxaliplatin + 50.4 Gy	ycT2N0	2	2	Positive	Required/Refused	32	LAR + IORT	61	Lung
61/M	T3N+	Capecitabine + 55 Gy	ycT0N0	3	3	Negative	Required/Refused	41	APR	53	Liver, lung
79/F	T2N+	Capecitabine + 50.4 Gy	ycT0N0	0	1	Negative	Not required	44	Refused	None	None
72/F	T3N+	Capecitabine +oxaliplatin +50.4 Gy	ycT2N+	0	1	Negative	Not required	41	CRT	45	Adrenal gland
63/M	T2N+	Capecitabine +55 Gy	ycT1N0	0	1	Negative	Not required	None	None	32	Liver, lung
54/F	T2N+	Capecitabine +oxaliplatin +50.4 Gy	ycT2N0	1	4	Negative	Performed	None	None	33	Lung
54/M	T2N0	Capecitabine +oxaliplatin +50.4 Gy	ycT3N0	0	1	Negative	Not required	None	None	101	Nodal, bone
54/M	T3N+	Capecitabine + 55 Gy	ycT3N0	2	3	Negative	Performed	None	None	9	Liver

*APR* abdominoperineal resection, *cTN* clinical tumor-nodal stage, *LAR* low anterior resection, *LAR* + *IORT* low anterior resection and intraoperative radiotherapy, *LE* local excision, *nCRT* neoadjuvant chemoradiotherapy, *TME* total mesorectal excision, *TTLR* time to local recurrence, *TTDR* time to distant recurrence, *ypT* post-neoadjuvant chemoradiotherapy pathological tumor stage

The estimated cumulative 5- and 10-year DRFS were 90% (95% CI 80–95), and 86% (95% CI 73–93), respectively (Fig. 2d). Eight (13%) patients had distant recurrences. Of eight patients experiencing distant recurrences, five died.

Overall, 49 (77.8%) patients had their rectum preserved, and 54 (84.1%) were stoma-free. The reasons for a definitive stoma were fecal incontinence after sphincter saving surgery (*n* = 1), rectal stricture after LE (*n* = 1), completion surgery after LE because of the presence of histopathologically unfavorable features (*n* = 5), and salvage radical surgery for local recurrence (*n* = 2).

At the last follow-up, 13 patients (20.6%) had died.

## Discussion

The aim of this study was to evaluate the long-term oncological outcomes of patients with rectal cancer who showed a major (complete or near complete) clinical response to neoadjuvant chemoradiotherapy and then underwent full thickness LE. The main findings of the study were that both OS and RFS were close to 90% at 5 years and close to 80% at 10 years; and that 78% of patients had the rectum preserved, and 84% were stoma-free. These findings are encouraging and seem to support the hypothesis that LE may represent a safe alternative to TME, offering comparable results, minimal morbidity and better functional outcomes.^12^,^14^,^15^,^19^

Although with shorter follow-up, several prospective studies have been published in recent years. The American ACOSOG Z6041 multicenter phase 2 trial recruited 79 patients who underwent LE after neoadjuvant chemoradiotherapy.^11^ At a median follow-up of 56 months, the estimated 5-year OS and DFS were 90.9% and 79.3%, respectively. As in our series, two out of five local recurrences were found in patients with a pCR. This is not surprising as the LE neither includes all the area of the pre-treatment primary tumor, nor removes the mesorectal nodes. Moreover, an incomplete histopathological examination cannot be excluded. In their prospective trial, Lezoche et al. randomized 100 patients who, after neoadjuvant chemoradiotherapy, underwent LE (*n* = 50) or standard laparoscopic TME (*n* = 50).^19^ At a median follow-up of 9.6 years, the cancer-related survival rate was 89% and the OS was 72%, without any differences compared with the laparoscopic TME arm. The rate of local recurrence was 8%. Although both previous trials included patients with favorable cases (small rectal cancer, clinically staged as cT2N0), the outcomes are comparable with our study. More recently, prospective studies included clinical T2-T3 rectal cancer.^14^,^15^ At a median follow-up of 60 months, Rullier et al. reported no difference between LE and TME arms, either in terms of 5-year local recurrence (7% vs. 7%), or in terms of metastatic disease (18% vs. 19%), OS (84% vs. 82%), or DFS (70% vs. 72%).^14^ Furthermore, at a median follow-up of 53 months, Stijns et al. reported a 5-year actuarial local recurrence rate of 7.7%, DFS of 81.6%, and OS of 82.8%, respectively.^15^ It should be noted that in our study, 60% of patients were staged as cT3 at baseline, whereas in the trials of Rullier et al. and Stijns et al. the rates of cT3 were 45% and 29%, respectively.^13^,^14^ These findings may suggest that the rectum preservation strategy should be based on clinical response to neoadjuvant therapy instead of clinical baseline staging. A clear message derived from our and the previous trials is that the risk of local recurrence after LE is higher than after TME surgery. In order to reduce this risk, patients with unfavorable histologic features, particularly ypT2-3 tumors, should undergo an early completion radical surgery. In three of five patients with local recurrences, the completion radical surgery was refused. Patients should be informed that LE is basically an excisional biopsy and that there is an increased risk of local recurrence, particularly for those patients refusing the recommended completion radical surgery (Table 3). Moreover, as all local recurrences were observed between 31 and 49 months after LE, a close and prolonged follow-up should be strongly recommended. This close follow-up is also required in patients with a pCR, as local recurrences have been observed in these patients.

Although this study reports on the LE approach, some considerations related to the watch-and-wait approach seem appropriate. Compared with the watch-and-wait policy, the LE approach is associated with postoperative morbidity and the need for completion TME, which is recommended in up to one third of cases,^8^,^14^,^15^ and may be challenging. On the other hand, LE provides a histological proof of pCR, avoiding the delayed diagnosis of regrowth; in these patients long-term impact on survival is matter or debate.^20^ In addition, while the watch-and-wait approach is only indicated in patients with clinical complete response, LE also seems appropriate in patients with a near complete clinical response. In the present study only 19 of 42 patients with a pCR were considered complete responders at restaging. Following the current indication (watch-and-wait to be performed only in patients with clinical complete response) 23 of 42 patients with pCR would have undergone TME instead of rectum preservation. An alternative approach could be to use both strategies within a rectum sparing program: watch-and-wait in patients with clinical complete response and LE in those with a near-complete clinical response. Independently from the strategy used, the key point still relies on the improvement of patient selection by better staging accuracy. As depicted in Fig. 1, in the group of patients with a pathologic major response (ypT0 and ypT1 with favorable histology) the rates of local recurrence were less than 5%, and more than 95% of patients were stoma-free with the rectum preserved.

The limitations of this study are related to the small sample size and to the lack of a comparative arm. Our institution is currently involved in a multicenter observational study (RESARCH), whose primary endpoint is to validate the rectal-sparing policy in patients with complete or near-complete clinical response after neoadjuvant chemoradiotherapy.^16^ Randomized trials in this field are challenging mainly due to the difficult accrual of patients. Likely, the best evidence may therefore derive from large observational prospective trials or national and international registry.^21^

## Conclusion

The present study confirms that, in patients showing major clinical response after neoadjuvant chemoradiotherapy, the LE approach may offer, in the long-term, similar encouraging survival outcomes to those previously reported in studies with shorter follow-up.^14^,^15^,^19^,^22^ Moreover, about 80% of patients could have their rectum definitively preserved.
